# Unveiling the Black Box of Diagnostic and Clinical Decision Support Systems for Antenatal Care: Realist Evaluation

**DOI:** 10.2196/11468

**Published:** 2018-12-21

**Authors:** Ibukun-Oluwa Omolade Abejirinde, Marjolein Zweekhorst, Azucena Bardají, Rudolf Abugnaba-Abanga, Norbert Apentibadek, Vincent De Brouwere, Jos van Roosmalen, Bruno Marchal

**Affiliations:** 1 Athena Institute Faculty of Science Vrije Universiteit Amsterdam Netherlands; 2 Department of Public Health Institute of Tropical Medicine Antwerp Belgium; 3 ISGlobal, Barcelona Institute for Global Health Hospital Clínic - Universitat de Barcelona Barcelona Spain; 4 Presbyterian Health Services-North Bolgatanga, Upper East Region Ghana; 5 Association of Church Development Projects (ACDEP) Northern Region Ghana; 6 Department of Obstetrics Leiden University Medical Centre Leiden Netherlands

**Keywords:** systems analysis, Ghana, clinical decision support, antenatal care, mHealth, program evaluation

## Abstract

**Background:**

Digital innovations have shown promise for improving maternal health service delivery. However, low- and middle-income countries are still at the adoption-utilization stage. Evidence on mobile health has been described as a black box, with gaps in theoretical explanations that account for the ecosystem of health care and their effect on adoption mechanisms. Bliss4Midwives, a modular integrated diagnostic kit to support antenatal care service delivery, was piloted for 1 year in Northern Ghana. Although both users and beneficiaries valued Bliss4Midwives, results from the pilot showed wide variations in usage behavior and duration of use across project sites.

**Objective:**

To strengthen the design and implementation of an improved prototype, the study objectives were two-fold: to identify causal factors underlying the variation in Bliss4Midwives usage behavior and understand how to overcome or leverage these in subsequent implementation cycles.

**Methods:**

Using a multiple case study design, a realist evaluation of Bliss4Midwives was conducted. A total of 3 candidate program theories were developed and empirically tested in 6 health facilities grouped into low and moderate usage clusters. Quantitative and qualitative data were collected and analyzed using realist thinking to build configurations that link intervention, context, actors, and mechanisms to program outcomes, by employing inductive and deductive reasoning. Nonparametric *t* test was used to compare the perceived usefulness and perceived ease of use of Bliss4Midwives between usage clusters.

**Results:**

We found no statistically significant differences between the 2 usage clusters. Low to moderate adoption of Bliss4Midwives was better explained by fear, enthusiasm, and high expectations for service delivery, especially in the absence of alternatives. Recognition from pregnant women, peers, supervisors, and the program itself was a crucial mechanism for device utilization. Other supportive mechanisms included ownership, empowerment, motivation, and adaptive responses to the device, such as realignment and negotiation. *Champion* users displayed high adoption-utilization behavior in contexts of participative or authoritative supervision, yet used the device inconsistently. Intervention-related (technical challenges, device rotation, lack of performance feedback, and refresher training), context-related (staff turnover, competing priorities, and workload), and individual factors (low technological self-efficacy, baseline knowledge, and internal motivation) suppressed utilization mechanisms.

**Conclusions:**

This study shed light on optimal conditions necessary for Bliss4Midwives to thrive in a complex social and organizational setting. Beyond usability and viability studies, advocates of innovative technologies for maternal care need to consider how implementation strategies and contextual factors, such as existing collaborations and supervision styles, trigger mechanisms that influence program outcomes. In addition to informing scale-up of the Bliss4Midwives prototype, our results highlight the need for interventions that are guided by research methods that account for complexity.

## Introduction

### Background

Digital health innovations have gained support as a means to improve health service delivery while strengthening health systems [1,2]. Mobile technologies (mobile health, mHealth) for maternal health in low-resource settings can play a role in addressing information, skills, and resource needs at various points in the continuum from prenatal to postnatal care [1,3,4].

The majority of digital health innovations for maternal health involve use of short messaging services, voice calls, point-of-care diagnostics, and health information management systems [1,4]. Other less explored areas recently gaining attention include its use for clinical decision support and remote monitoring. This is particularly important in the context of poor road networks, remote geographical locations, weak referral chains, and alarming workforce shortages. Diagnostic and decision-support systems are a group of digital health innovations that aim to address challenges of timely and effective health care, using evidence-based principles [2]. Despite evidence of their importance for task shifting and promoting adherence to clinical practice guidelines, attempts to embed them into large-scale service structures are yet to be attained [4].

Evidence on mHealth has been critiqued for being a black box with little knowledge from pilot projects to inform prototype development and scale-up [5]. The dominant discourse is that low technological skills alongside infrastructural barriers are at the root of poor mHealth uptake in low- and middle-income countries (LMICs). An alternative and less explored explanation is that factors unique to the ecosystem of health service delivery need to be accounted for, motivating calls for knowledge on mHealth that is grounded in theoretical understanding [6-8]. A recent theory-based analysis on what works or not for mHealth in maternal health service delivery has shown that LMICs are still at the adoption-utilization stage [9]. In their review, Chib et al also highlight a knowledge gap on mechanisms for mHealth adoption and the role of theoretical explanations in addressing these gaps [8].

This study aims to identify causal factors underlying the variation in mHealth usage during the adoption and utilization phases of an intervention and understand how to overcome or leverage these in subsequent implementation cycles. Findings will contribute to the body of evidence on contextual and domain-specific applications of similar innovations in other low-resource settings.

### Description of the Intervention

In 2016, a consortium of 7 organizations representing a south-north public-private partnership embarked on a project to prove the viability of a modular integrated diagnostic kit tagged the Bliss4Midwives (B4M) device (unpublished data [10]). The B4M device supports instant informed diagnosis during antenatal care (ANC) by enabling noninvasive point-of-care screening for preeclampsia, gestational diabetes, and anemia—3 main screening components of ANC. The components of the device include a noninvasive hemoglobin reader with infrared sensors mounted on a finger clip, a self-inflating blood pressure cuff, and an automated urinary dipstick reader for measuring urinary protein and glucose. In the absence of B4M, target beneficiaries in remote areas would otherwise have to travel to other health facilities to conduct these tests, delaying timely detection and management of high-risk complications [11]. B4M was introduced in 7 health facilities in the upper east region and northern region of Ghana. Additional details on the device, project setting, viability, and beneficiary experiences have been reported elsewhere (unpublished data [10];[11]).

Although both users and beneficiaries valued B4M, results from the pilot showed wide variations in usage behavior and duration of use across project sites (unpublished data [10]). Beyond establishing viability of the intervention, application of a theory-based approach requires assessing why and how exactly it works [12]. In line with the long-term goals of the consortium, evaluation findings will inform the design and implementation of an adapted B4M prototype.

## Methods

### Study Setting

A total of 6 prototype devices were deployed in 7 predominantly rural locations—4 facilities in the upper east region and 3 in the northern region. A total of 25 maternal health workers were trained to operate B4M. As the device was withdrawn from 1 facility in the second month of the intervention, the evaluation focused on 6 of the 7 health facilities: facilities A to D in the upper east region and facilities E and F in the northern region. Facility A is the ANC unit of a district hospital and the first-level referral point for facilities B, C, and D, which are health centers. Facility E is an independent public health unit of a district hospital, whereas F is a health center. With the exception of facilities B and C, which shared a single B4M device on a rotating schedule, the other facilities had stable access to 1 device each.

**Table 1:** Adoption and utilization per health facility.

Adoption level/Utilization level	Low adoption	Moderate adoption	High adoption
Low utilization	Facilities B and E	Facility C	Facility A
Moderate utilization	N/A^a^	Facility F^b^	Facility D
High utilization	N/A	N/A	N/A

^a^N/A: not applicable.

^b^Due to data loss and inability to track the usage trend in facility F, we relied on cumulative usage data and reports from monitoring visits.

**Table 2:** Clustering of cases.

Usage combinations	Clusters^a^		
	Low usage	Moderate usage	High usage
Low adoption—low utilization	Facilities B and E	N/A^b^	N/A
Moderate adoption—low utilization	Facility C	N/A	N/A
Moderate adoption—moderate utilization	N/A	Facility F	N/A
High adoption—low utilization	N/A	Facility A	N/A
High adoption—moderate utilization	N/A	Facility D	N/A
High adoption—high utilization	N/A	N/A	None

^a^As utilization covered a longer period than adoption and total duration of use varied between facilities, when defining clusters, cases were stepped down to account for this.

^b^N/A: not applicable.

### Study Design

We employed a multiple case study design, defining a case as 1 B4M health facility [13]. Informed by knowledge of the project, ANC volume per facility and trend analysis on adoption (first 2 months) and utilization (continued or prolonged use over time) of the device over a 10-month period (unpublished data [10]), health facilities were classified as low (average number of screenings <15 per month), moderate (average number of screenings ≥16 and ≤40 per month), or high (average number of screenings ≥41 and ≤75 per month) adoption and utilization (Table 1). Cases were subsequently grouped into 3 usage clusters: low, moderate, and high, whereby the term usage is a composite term describing adoption and utilization (Table 2). No health facility fell under the high usage cluster, which was recognized as the ideal state. The evaluation sought to understand usage variation between low and moderate usage clusters and reflect on how a high usage state may be attained in implementing an improved prototype.

### Evaluation Methodology

Realist evaluation is a theory-based approach for opening the black box on complex interventions [14,15]. It has shown promise in unraveling explanations for complex interventions in health, international development, and technological innovation [16-18]. It involves an iterative process beginning and ending with program theories, systematically moving from the specific to the abstract, described as “climbing the ladder of abstraction” [19,16]. Realist methodology is suited for evaluating B4M because it is method neutral and can aid an in-depth understanding of the explanatory processes for program outcomes as well as in the identification of implicit and explicit mechanisms underlying them.

Due to its theoretical underpinning and applicability in real-life settings, realist methodology was applied to assess differences between low and moderate B4M usage clusters. This involved developing and subsequently testing initial program theories using qualitative and quantitative data. Identified causal explanations underlying variation in mHealth usage between clusters were framed in configurations that showed the interrelationship between the *Intervention*, implementation *Context*, participating *Actors*, explanatory *Mechanisms*, and *Outcomes*. Simply put, *ICAMO* configurations. Using this analytical heuristic, 2 main layers of context may be differentiated: the broad external environment in which interventions are situated (C_1_) and the health system or health facility setting in which mobile technology is introduced (C_2_). Where mechanisms broadly refer to the reasoning and responses to the B4M intervention underlying observed outcomes, main mechanisms (M) were differentiated from subexplanatory mechanisms (m).

**Figure 1:**
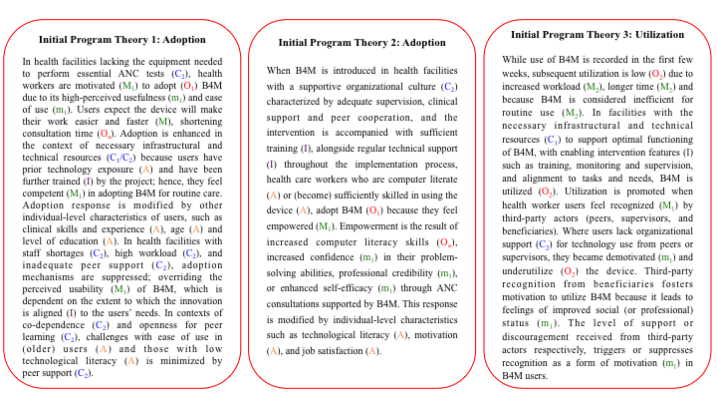
Initial program theories. Features and characteristics of the intervention- (I); Contextual factors are denoted (C_1_) and (C_2_) for environmental and health system context respectively; Outcomes are denoted (O_1_) or (O_2_) representing adoption and utilization respectively; Mechanisms are identified (M_1_) or (M_2_) following the outcomes they are linked to, with related explanatory mechanisms further depicted (m_1_) or (m_2_); Actor or user characteristics are denoted (A); (O_a_) represents additional outcomes. ANC: antenatal care; B4M: Bliss4Midwives.

### Initial Program Theories

The initial program theories of the B4M intervention, which describe how the intervention was expected to work, were developed using a 2-pronged approach:

- A realist review of how mHealth influences performance of maternal health workers in LMICs was conducted. A total of 4 factors necessary for the successful adoption and utilization of mHealth were identified: general environmental context, organization of the health system, intervention factors, and individual factors [9].
- To ensure that the initial program theories were aligned to the unique prescription of the B4M intervention, we refined literature-based theories by analyzing the research protocol and interim progress reports. We also conducted a focus group discussion and follow-up interviews with members of the program consortium, resulting in 3 initial program theories (Figure 1). These processes informed data collection tools and guided analysis.

### Data Collection

Using quantitative and qualitative methods, the 3 candidate initial program theories were empirically tested. Data collection activities are presented in Multimedia Appendix 1 and summarily involved:

- A total of 24 semistructured interviews with device users, health facility managers, local program managers, and district health information officers trained to provide technical support. Interviews were conducted in English and lasted between 22 and 122 min (mean=60 min).
- A total of 14 usability questionnaires measuring perceived usefulness and ease of use of B4M using 12 items each, developed from standardized tools [20,21] and administered to device users (Multimedia Appendix 2). Respondents selected options from strongly disagree (1) to strongly agree (5) on a 5-point Likert scale, totaling 12 to 60 points per construct.
- Health facility checklists at 6 facilities, to assess their capacity to provide ANC services, referral, or management of emergencies (Multimedia Appendix 3). Observation of ANC service provision was conducted in 5 facilities.
- A focus group discussion with project implementers.
- A theory-validation meeting with 16 B4M users.

All interviews and meetings were conducted in English, audio recorded, and transcribed verbatim.

### Data Analysis

For the data on usability, negative statements were reverse coded, and raw scores were exported to SPSS. Nonparametric *t* test was used to compare perceived usefulness and perceived ease of use of B4M between clusters. Interview transcripts as well as observation and field notes were analyzed using realist thinking, applying an interpretive lens to build a casual web of explanations from multiple strands of evidence [22]. Using abductive inference, we started from the main outcomes of interest (adoption and utilization) and *worked backward* to trace plausible underlying explanations. We queried the data for mechanisms of perceived usefulness, perceived ease of use and empowerment (self-efficacy and confidence) for adoption, and the mechanism of recognition for utilization, while being open to new configurations.

A cumulative stepwise approach applying inductive and deductive reasoning was employed. First, aided by an Excel spreadsheet, we entered information on each health facility that *spoke* to elements of the ICAMO configuration into rows and columns, including supporting quotes. Furthermore, previous analysis has shown that over time, the intervention itself can become a new contextual layer within the study setting [9]. Nevertheless, we chose to differentiate the intervention (I) from the existing contextual factors (C_1_ or C_2_) to clarify the resources and support that are specifically introduced by B4M. As our data were closer to the project itself than to the broader environmental context (C_1_), we did not have sufficient strands of evidence on this level. Next, the realist thinking of “if C, then O, because M, for A” was applied to develop ICAMO configurations for each cluster. This involved grouping similar patterns and corroborating or voiding strands of preliminary evidence. Although most evidence strands manifested to varying degrees in each facility, when these were not sufficient to explain usage behavior, they were discarded from the configuration. Theory testing and refining were incremental; data from the low usage cluster were first assessed and then compared with data from the moderate usage cluster. Finally, a cross-case comparison between clusters was used to develop refined program theories.

### Ethical Considerations

Study approval was granted by the Navrongo Health Research Centre Institutional Review Board (approval ID: NHRCIRB18) and the EMGO+ Scientific Committee of the Amsterdam Public Health Institute (reference number: WC2017-026). Before all interviews, written consent was secured using informed consent forms.

## Results

### Usability Statistics

Respondents’ characteristics and usability scores are presented in Multimedia Appendix 4. Acknowledging individual variations, the perceived usefulness and perceived ease of B4M use were relatively high in all facilities (range 39.0-58.0). The *t* test showed no statistically significant differences between the 2 usage clusters (Table 3).

**Figure 2:**
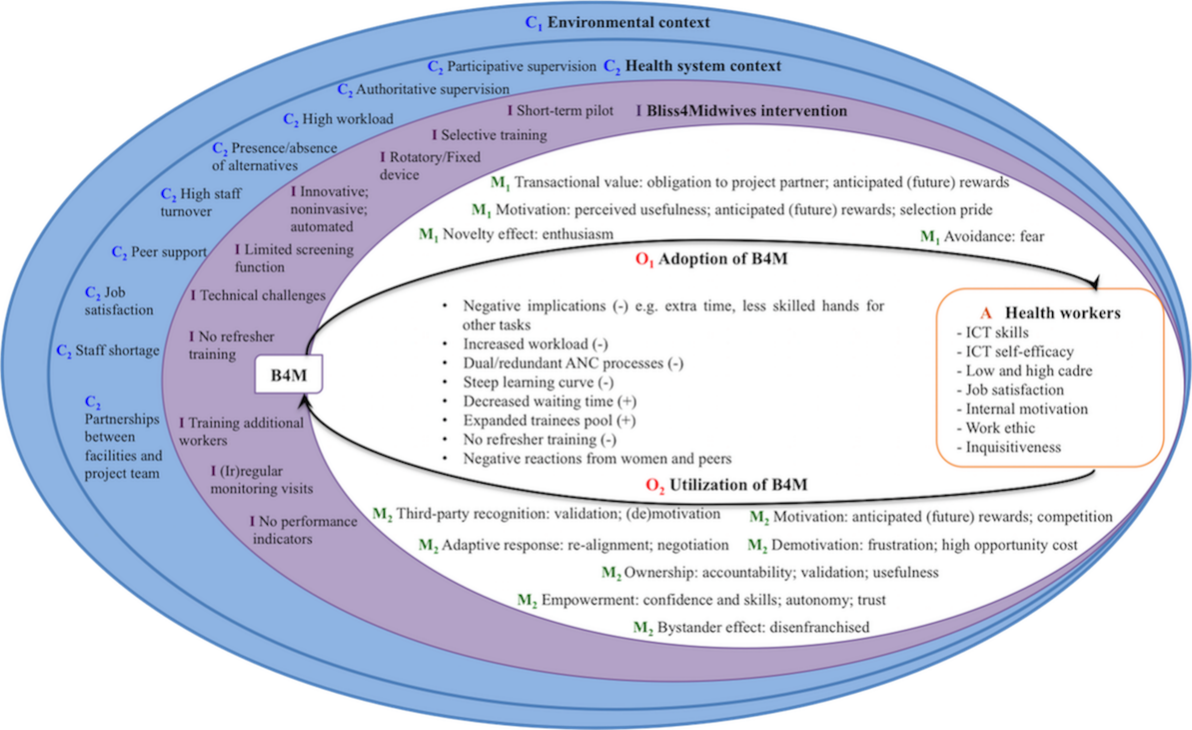
Summary of findings. Ecosystem of ICAMO factors underlying the adoption (O_1_) and utilization (O_2_) of B4M within a complex context (concentric circles C_1_ and C_2_) and features of the B4M intervention (I). M_1_ and M_2_ are mechanisms related to outcomes O_1_ and O_2_ , mediated by user characteristics (A). Bullet points highlight other facilitating (+) or inhibitory (–) factors influencing usage behavior. ANC: antenatal care; B4M: Bliss4Midwives; ICT: information and communication technology.

Next, we present the refined program theories under each outcome of interest in narratives of ICAMO configurations. Intervention features are marked “(I)” factors related to the health system context as “(C_2_)” evaluation outcome “(O_1_)” represents adoption and “(O_2_)” utilization, whereas “(O_a_)” represents additional outcomes. Mechanisms are identified “(M_1_)” or “(M_2_)” following the outcomes they are linked to, with related explanatory mechanisms further marked “(m_1_)” or “(m_2_).” Actor or user characteristics are marked “(A).” Explanations are included in the narrative, noting differences between cases and usage clusters. An overview of the realist analysis is depicted in Figure 2.

### Adoption (O _1_)

Adoption of B4M was characterized by an initial upward climb in both clusters. Differences, however, stemmed from experienced technical failures (I), complete or partial presence of an alternative point-of-care device or onsite laboratory (C_2_), and dispositions of individual users (A). In health facilities with limited capacity to perform basic ANC screening tests (C_2_), trained midwives and community health workers (I) were enthusiastic (m_1_) to adopt B4M (O_1_). This was due to its novelty (M_1_) as a noninvasive automated device (I) and in anticipation of service delivery benefits, which they considered important (m_1_) for providing focused ANC:

After training, we were just eager [...] If we don’t support whatever the project’s intention is, it will not be realised. Then it means the support we could have also gotten from it will not come.

Facility C

In facilities A, E, and F, long-standing relationships with local project partners (C_2_) played a role in their selection as project sites (I). Their adoption response was transactional (M_1_), triggered by a sense of obligation (m_1_) to the project partner and by pride from being selected (m_1_). Where alternative screening options were not functionally reliable (C_2_), were not trusted (C_2_); as was the case in facility F, required a longer turnaround time (C_2_); as in facility A, or when screening was a paid service (C_2_), users were motivated (M_1_) to adopt B4M (O_1_). This is because they considered it to be a necessary alternative (m_1_), a trustworthy expert (m_1_), a time-efficient resource (m_1_), and a cost-effective substitute (m_1_):

Supposing I am here alone, I can’t talk to anybody…the machine can tell me what to do [...] So, it is more accurate to use it.

Facility F

**Table 3:** *t* test for equality of means on usability assessment.

Construct	Low usage cluster^a^, mean (SD)	Moderate usage cluster^a^, mean (SD)	*P* value
Perceived usefulness	52.0 (4.6)	47.0 (6.2)	.11
Perceived ease of use	50.4 (7.1)	51.4 (8.1)	.81

^a^*P*<.05.

Irrespective of age, baseline technological skills, years of clinical experience, and the additional time use of B4M required, users with high technological self-efficacy (A) or willing to acquire technological skills (A) adopted the device despite an initial (1-2 weeks) difficult learning curve. Workers with low technological self-efficacy (A) avoided B4M (M_1_) out of fear (m_1_) of damaging it and low effort expectancy, although they reported sufficient training (I) and encouragement from peers and the project (C_2_). To fill training attrition gaps due to user disinterest (A) and staff turnover (C_2_), additional training (I) was organized for other health workers, thereby expanding the pool of trainees (O_a_) who were mostly younger, lower cadre personnel (ie, community health nurses and nursing assistants who are trained professionals):

So, when I went for the training on the kit, it was a hell with me and the nurses [...] I don’t know the thing and I don’t want to make mistakes [...]We have people who know the thing, so why should I be forcing my head to be doing all these things, when these young girls are sitting there?

Facility E

### Utilization (O _2_)

In response to contextual and program factors, postadoption utilization of B4M was explained by the dominance of either suppressive or supportive mechanisms triggered at facility and individual levels. Supportive mechanisms identified in the moderate usage cluster were less prominent or lacking in the low usage cluster.

#### Implementation Strategy: Fixed Versus Rotatory

The rotating strategy (I) between facilities B and C (from the low usage cluster) required ownership of the rotation process (M_2_) and necessary resources (C_2_) including fuel and motorbike. Although facility B had more resources, users in facility C demonstrated higher ownership—using the device more consistently when present. As ANC schedules between rotatory sites sometimes overlapped (C_2_), the device was often absent from points of need or present without use. More importantly, the rotation strategy not only affected the number of screenings in facilities B and C but also whether a woman repeatedly benefitted from its use throughout pregnancy:

It wasn’t that (convenient), [...] (sigh) because some women, you have used it on them and then when they come back, it is not there. The next time, it becomes a problem.

Facility C

All facilities in the moderate usage cluster had a fixed device (I), and health workers quickly gained dexterity (O_a_), when they felt supported by supervisors and peers (C_2_), alongside other enabling contextual factors.

#### Empowerment

Health workers felt empowered (M_2_) by B4M in 2 ways. First, users increasingly gained confidence and skills (m_2_) in device use and ANC referral, shortening the time needed per screening (O_a_). In lower cadres who did not know what to do (C_2_) and higher cadres (ie, trained professional midwives) who overlooked warning signs due to work pressure (C_2_), B4M was used to validate hunches and keep users alert (I) because they trusted its accuracy (m_2_). Second, both facilities with and without alternatives experienced more autonomy (m_2_) and a decreased need for diagnostic referrals, which previously delayed the care cycle:

Even though you are experienced and you know what to do, you may be tired or distracted, so the device will not allow you miss a critical case.

Facility D

Users with low technological skills (A), without refresher training (I), in facilities with a rotatory implementation (I), or inconsistent usage (O_a_) were demotivated (M_2_) and frustrated (m_2_) because they frequently forgot how to navigate the system (O_a_), contributing to nonpartial or partial use (O_2_). By limiting access (I), the rotating strategy effectively suppressed the empowering effect of B4M:

When you don’t use it for some time, you forget.

Data validation meeting

#### Realignment and Negotiation

Misalignment of the device (I) to existing work processes or limited workspaces made usage frustrating (m_2_). Evidence from the moderate cluster showed that if trainees felt compelled or were otherwise motivated by current or anticipated benefits to use B4M, their adaptive response (M_2_) was realignment (m_2_) or negotiation (m_2_). In the low usage cluster, the response was rejection and abandonment (m_2_):

It was cumbersome because of our setting. Where to put (the device) was a problem [...] It was really hindering us, because who would be standing and doing all this?

Facility E

Oh, it is an interruption but since we’ve been able to manage it, it is no more an interruption again.

Facility D

Realigning workflow as a coping mechanism to B4M involved peer-training other (lower cadre) health workers (O_a_) who showed keen interest and were inquisitive (A). Peer-trained users (A), however, had low confidence (m_2_) in the thoroughness of training, manifesting low ownership (M_2_). Realignment allowed for redistribution of roles (O_a_) with at least two workers conducting ANC when the device was in use. This meant that midwives could focus on core maternal health tasks (palpation, deliveries, and counseling), whereas lower cadre staff operated the device. This strategy was not feasible in contexts where support staff had other fixed duties such as outreach visits (C_2_), where only 1 midwife was available per time (C_2_), or in contexts of high staff turnover or B4M-training attrition due to administrative leave or transfer (C_2_). In facility D, users did not only manage their own expectations and avoid dual use of screening options but actively negotiated (m_2_) B4M usage with beneficiaries (O_a_):

You know, when human beings tune their mind to something, they expect only that. I told them that the machine will have to check everything for them and it will tell us what to do [...] In fact, now, we don’t talk about it. When they come, everybody is relaxed.

Facility D

In contexts of professional isolation (C_2_), low (supervisory) recognition (C_2_), low job satisfaction (C_2_), and high workload (C_2_), it was not sustainable for users to persevere against all odds, which manifested in low utilization (O_2_).

#### Opportunity Cost and Competitive Edge

Facilities with high volumes of ANC attendees (C_2_), multiple service delivery demands (C_2_), or high staff turnover or shortages (C_2_) manifested suboptimal utilization of B4M (O_2_) despite high perceived usefulness (m_2_) and high perceived ease of use (m_2_). This was linked to the demotivating (M_2_) high opportunity costs (m_2_) of usage, including the following: (1) ANC consultations took longer; (2) B4M did not completely remove the need for diagnostic referral for other tests; and (3) B4M was used in addition to the usual ANC routine because it was regarded as a pilot intervention. Where B4M represented a partial solution (I) to a larger diagnostic need and was not fully integrated (I) into ANC workflow, duplication of processes made utilization burdensome (m_2_), causing dissatisfaction (M_2_) and decreased perception of its usefulness (m_2_):

It’s easy to do either the standard or B4M. It’s the combination that is not easy [...] It helps you to waste a lot of your time. It’s like the thing became not useful to us again.

Facility F

Health workers in moderate usage facilities took ownership (M_2_) of the device and utilized it because of their strong work ethic (A), motivation (M_2_) to meet service delivery needs, and expectation of appreciation (m_2_) at project end. To defend their professional image and as a favor to their local program managers, these users had an internal drive to compete (m_2_) and perform better than other facilities:

What I can say about the midwives here is that we take our work serious [...] Sometimes there are certain things you don’t want to do, but when it comes to our work anything we have to do we do it.

Facility F

If users believed that project success and subsequent reward were based on the number of screening records per facility, utilization was higher, with less regard to follow-up screening of beneficiaries at each visit (O_a_). Absence of project feedback on performance indicators (I) and lack of direct incentives (I) suppressed (in the low cluster) and dampened (in moderate cluster) the competitive edge:

So we needed that they should tell us that what we were doing is actually an important thing [...], then we would put our efforts in getting the whole thing done properly.

Data Validation Meeting

#### Third-Party Recognition

We found that recognition from third-party actors (M_2_) as a form of external motivation was an important mechanism underlying utilization, and this derived from multiple sources: (1) peers who supported and encouraged device use, (2) pregnant women who projected the value of the device to their trust in the health worker, (3) program staff who provided technical support and conducted monitoring visits, and (4) supervisors at facility and district levels. In facilities A and D, peers regarded B4M users as distinguished, belonging to an expert niche. This sometimes increased utilization motivation (m_2_), but in many other cases, it caused tension (m_2_) when peers felt that trainees had enjoyed preferential selection and benefits from the intervention. Peers, therefore, tagged B4M users as lazy or unserious:

I feel proud (when my colleagues call me computer woman).

Facility D

The perception is worse about you who went and learnt because you can now (do these things). But the thing is that you went and signed and took money (ie, participation and per diem during B4M training).

Data Validation Meeting

B4M users felt respected by pregnant women who showed increased confidence in health workers’ professional credibility (m_2_), especially in lower cadre workers (A). However, the comparatively longer time (I) it took compared with the standard ANC routine elicited negative reactions (m_2_) manifested in body language or grumbling from pregnant women. This demotivated (m_2_) users and led to decreased utilization (O_2_):

Sometimes, the women think that you are doing it for them and so that kind of trust comes in [...] They are happy that it is madam midwife who is doing it for me, but not necessarily the bliss for midwife that is doing it. So, it sort of gives you that zeal to continue using it.

Facility C

Irregularity of monitoring visits (I) and technical problems (I) led to prolonged periods of nonuse (O_2_) because users forgot (m_2_) about the intervention and no longer considered it a priority (m_2_). Due to easy geographical access (C_2_) and strong preintervention collaboration (C_2_), facilities A and D from the moderate cluster frequently received monitoring visits (I) from the project manager, which kept users *on their toes* (m_2_) and stimulated ownership (M_2_). It also made users feel validated (m_2_) and not exploited by the project to extract usage data:

We didn’t expect to see money. Money could be one of the things, but regular visits, calls and all those things; we were not getting it at all. So we just said “Aha, so the person just comes to take the (data) and goes away.”

Data Validation Meeting

In facilities where workers feel unsupported by superiors (C_2_) and where aspirations for career progression and professional development are not fostered (C_2_), users were demotivated (m_2_) and did not take ownership (M_2_). The project, therefore, became a platform to silently protest job satisfaction through nonuse:

But sometimes it’s really heart-breaking. Like why should I really waste my time doing this and at the end of the day nobody appreciates you? Even what we are supposed to work with is not there.

Data Validation Meeting

#### Ownership and Supervision Styles

Ownership (M_2_) of device usage trickled down to users from higher-level actors at program, district, and facility levels, based on supervision styles (C_2_). If authority figures did not demonstrate the importance of B4M, health workers were less inclined to use the device because they did not feel accountable (m_2_) and felt discouraged and unappreciated (m_2_). Authority figures in the moderate usage cluster showed more engagement with the program.

In facilities with firm hierarchical structures such as facility A, where users were accustomed to authoritative supervision (C_2_), involvement of a high-ranking supervisor (C_2_) imposed accountability and responsibility (m_2_), reinforcing device use. In facility D, on the other hand, ownership was fostered by supportive participative supervision (C_2_) in motivated health workers with high self-efficacy (A) in using technology:

Because it came and our matron called and said “I’m putting this thing in your hands, take care of it.” So, because it was from her, we were doing it [...] And often the matron would come and ask “Are you people with the box? Are you ok?” Then the next day, again. So if you are not there and she comes and the box is lying there, there would be problem. So, we are always doing it.

Facility A

#### Bystanders and Champion Users

By training only a select number of staff in each facility (I), the project could not leverage collective ownership at facility level and some users felt unsupported by disenfranchised peers (m_2_). Even when multiple persons were trained (I), in contexts of low-shared responsibility (C_2_) and weak interpersonal relationships (C_2_), a bystander effect (M_2_) was observed. As seen in moderate usage facilities, responsibility for B4M was indirectly delegated to a *champion* user (A) who had strong internal motivation (A) and in whose absence (C_2_) the device was not used (O_2_). Nevertheless, given other competing priorities (C_2_) and to balance the inconvenience of using the device, usage was restricted to 1 day a week or to a few hours in a day:

Yea, at first, the excitement was just too much. But when I trained this lady and she picked it very fast, then I stopped using it.

Facility D

If she is there, nobody will even tell her what to do. [...] I used the word passionate—if you have the zeal to work. For her, I think no matter the pressure she can be called at any time and she won’t have any problem, unlike some others.

Facility F

## Discussion

### Overview

The theoretical bases of knowledge on adoption and postadoption have been largely developed in the field of management information systems with a focus on higher-income countries [23,24]. As digital innovation systems continue to expand in LMICs, the implications of these theories in low-resource settings such as Ghana are making their way into the research agenda [8,25,26]. To our knowledge, this is the first study that applies a realist lens to elicit theory-based explanations on mHealth for maternal health services in LMICs. Our analysis confirmed some components of the initial program theories, voided others, and unveiled additional elements previously unaccounted for. Below, we reflect on key findings and their relevance to the science of mHealth implementation.

### Principal Findings

In facilities with limited diagnostic capacity, motivated workers adopted B4M for its novelty and benefits, in contexts of existing collaborations and authoritative or participative supervision styles. Although technology novelty triggered supportive adoption mechanisms, we found that the actual utilization of the device was the most important phase of the usage cycle [23]. Above-average usability scores from most health facilities did not fully explain variation between usage clusters, confirming the disconnect between usability and actual use [27]. Fear, enthusiasm, and high expectations for service delivery, especially in the absence of alternatives, better explained low to moderate adoption of B4M. With increased experience of use, we found that the initial emotive adoption response was replaced by rational behavior in the utilization phase: perceived usefulness being overshadowed by experienced contextual difficulties. Saccol and Reinhard describe this contrast between the perceived magic of technology and the disappointment of its limitations in the real world, which dampens users’ initial enthusiasm [28]. Although the program designers’ expectation was that all or most facilities would operate under the high usage cluster, that is, high adoption followed by high utilization, the identified supportive or suppressive mechanisms within and between cases shed light on why no health facility fell under this ideal state.

Realignment of mHealth to workflow and beneficiary expectations of ANC was identified as a crucial adaptive mechanism for its utilization. In addition to intrinsic motivation and a sense of accountability in users, utilization was influenced by mechanisms triggered in third-party actors. Negative reactions from pregnant women, bystander effect in peers, and low support or ownership from supervisors and program managers caused low utilization. mHealth adoption has been described as a social process [29], which may explain the strong third-party effect, although its influence has been specifically linked to contexts of mandatory technology use [30]. Despite perceived usefulness and user motivation, utilization mechanisms were suppressed by intervention-related (technical challenges, device rotation, lack of performance feedback, and refresher training), contextual (staff turnover, high workload, competing priorities, and low job satisfaction), and individual (low technological self-efficacy and knowledge) factors. Champion users displayed moderate but inconsistent adoption-utilization behavior, by taking ownership of the device, defying usage barriers. This adaptive behavior of users as a distinguishing factor in usage behavior is in line with other studies [27].

Contrary to the expectation that usage behavior was related to age, we found that internal and external motivations and technological self-efficacy were stronger explanatory factors. However, these are linked to age as a predictor of technology usage. Previous research confirms that older users have lower technological self-efficacy and are intimidated by the steep learning curve, especially when they have low baseline technological skills and inadequate learning support [31-33]. Although we found that empowerment was triggered in the utilization phase, adoption behavior has been shown to predict utilization response [34]. This mechanism might, therefore, manifest in both phases.

### Implications for Bliss4Midwives Prototype II and Other Mobile Health Interventions

Beyond initial training, introducing technology requires careful planning and adaptation in low-resource settings where not many users experience job satisfaction or have adequate technological training as part of their professional competencies [35]. Admittedly, most factors related to the intervention context and actors such as service delivery demands, workforce shortages, and staff turnover are beyond the control of implementation teams. Nevertheless, these will have to be constantly negotiated especially in the utilization phase, with a responsive implementation strategy that supports workflow alignment and integration, which are crucial to the success of mHealth [35,36]. A preintervention situation analysis that takes our findings into account would go a long way in ensuring that future interventions are holistic and context-specific. A practical starting point to this could involve incorporating ICAMO elements into applicable implementation research frameworks such as the Consolidated Framework for Implementation Research, which incorporates multilevel factors and is adaptable through program cycles [37].

The temporal nature of pilot projects imposes a false sense of reality. Although users may briefly accommodate the innovation, they will be less invested in making long-term commitments requiring individual and organizational realignment, for short-term gains. In addition to being user-centered and accounting for the context, it is imperative that multiple stakeholder perspectives are leveraged during innovation design [6,7,35]. High-ranking supervisors might seem distant from the usage process but could compel or foster accountability and usage. They can also support adaptive strategies to integrate technologies into routine practice, especially in contexts of hierarchical supervision [30,38].

Selective training of a few workers unintentionally limits collective ownership and accountability for usage behavior. All health workers involved in maternal health service delivery at each site should be trained on device use, with regular monitoring and supervision, and periodical refresher training to help sustain or improve technological self-efficacy and dexterity, consequently preventing frustration and utilization decline [39]. Closer supervision and attention will be necessary in users with lower baseline technological skills and self-efficacy. Although it may cause tension and resistance from higher cadre users or peers, workers who fit the typology of champion users should be identified and encouraged to serve as opinion leaders within their health facilities. This would improve collective ownership, minimizing the bystander effect and optimizing social pressure [39,40].

The value of preexisting collaborations between the local partner organizations and health facilities and other administrative bodies remains crucial to gain access and influence, motivate, and encourage users. However, sustainability of transactional responses as a favor to program managers is doubtful. Financial incentives as a mechanism for behavior change have elicited mixed reports [41]. Indirect incentives such as encouragement, recognition, and support, which were highly desired and valued by B4M users, can, however, be promoted. To leverage the competitive mechanism and give users regular performance feedback, respectively, the design features of B4M prototype II could include gamification and dashboard analytics.

### Limitations

At the time of data collection, the device was in limited use in 3 of the 6 sites, with some respondents reporting not using the device for up to 5 months. This introduced recollection bias, in addition to socially desirable answers. Furthermore, not all user experiences were captured because a small number of trained users were unavailable for interviews. By triangulating data from multiple sources and interviewing at least two users per site, we attempted to compensate for these. The data validation workshop and dissemination meeting also informed group consensus on our findings. A realist approach is best applied throughout the life cycle of a project, from design to evaluation and reporting [22]. The nature of B4M as a short-term pilot, in addition to other constraints, restricted this possibility. Nevertheless, by developing and testing 3 initial program theories, the refined theories as a result of our analysis are sufficient for the next phase of prototype development.

### Conclusions

This study shed light on optimal conditions necessary for B4M to thrive in a complex social and organizational setting. Evidence on the growth and potential of mHealth in improving service delivery, especially in a critical domain such as maternal health, may have overshadowed important individual, health system, and implementation factors that preclude its alignment in specific contexts and by certain user types. Beyond usability and viability studies, advocates of innovative technologies for maternal care need to consider how contextual factors, such as existing collaborations and supervision styles, trigger supportive mechanisms that influence program outcomes. This knowledge can be used to design and implement mHealth in similar settings. In addition to informing scale-up of the B4M prototype, our results and approach highlight the need for interventions that are guided by research methods that account for complexity.

## Appendix

Multimedia Appendix 1 Data collection activities.

Multimedia Appendix 2 Usability survey questionnaire.

Multimedia Appendix 3 Health facility checklist for antenatal care (ANC) service provision.

Multimedia Appendix 4 Characteristics of Bliss4Midwives (B4M) users and usability scores.

## Abbreviations

ANC: antenatal care
B4M: Bliss4Midwives
ICAMO: intervention-context-actor-mechanism-outcome
LMICs: low- and middle-income countries
mHealth: mobile health
