# Supplementary material for: Unveiling the Black Box of Diagnostic and Clinical Decision Support Systems for Antenatal Care: Realist Evaluation
Source: JMIR Mhealth Uhealth. 2018 Dec 21;6(12):e11468. doi: 10.2196/11468 (PMC6320439; doi:10.2196/11468)
Supplement: Multimedia Appendix 1 [file mhealth_v6i12e11468_app1.pdf]

Multimedia Appendix 1

Data collection activities and Respondents

	Interviews	Health Facility survey	B4M User survey	ANC observations*	B4M observations
**Upper East Region**	- 1 program manager<br>- 1 district health information officer<br>- 1 district health director	N/A	N/A	N/A	N/A
Facility A	- 3 health workers<br>- 1 health facility manager	1	3	Yes	Yes
Facility B	- 2 health workers<br>- 1 health facility manager	1	2	Yes	Yes
Facility C	- 3 health workers<br>- 1 health facility manager**	1	3	No	No
Facility D	- 2 health workers<br>- 1 health facility manager**	1	2	Yes	Yes
**Total**	**15 respondents**				
**Northern Region**	- 1 program manager<br>- 1 district health information officer	N/A	N/A	N/A	N/A
Facility E	- 3 health workers;<br>- 1 health facility manager	1	2	Yes	No
Facility F	- 2 health workers<br>- 1 health facility manager	1	2	Yes	No
**Total**	**9 respondents**				
**Mixed**	- FGD with 5 consortium members	N/A	N/A	N/A	N/A
	- Data validation meeting with 16 B4M users	N/A	N/A	N/A	N/A
	**21 respondents**				

* B4M device was not always in use during ANC observations even when present in the facility

** Health facility manager was also a B4M user
