# Supplementary material for: Unveiling the Black Box of Diagnostic and Clinical Decision Support Systems for Antenatal Care: Realist Evaluation
Source: JMIR Mhealth Uhealth. 2018 Dec 21;6(12):e11468. doi: 10.2196/11468 (PMC6320439; doi:10.2196/11468)
Supplement: Multimedia Appendix 2 [file mhealth_v6i12e11468_app2.pdf]

Multimedia Appendix 2

BLISS4MIDWIVES (B4M): QUESTIONNAIRE ON PERCEIVED USEFULNESS & EASE OF USE

(Bring out printed color-coded sheet for likert scale and explain the colors to respondent. Ask respondent to point to which square/color applies for each statement that you make. Tick the appropriate box in the survey sheet. Following responses, probe further before moving to the next question)

Date of Interview …………………. Location ……………………… Interviewer Initials …………...

Health Facility: Name……………………………………….. District …………………………….

Initials of Midwife/CHN …………...…………. Assigned Project Code …………………………….

Confirmation of Consent:(Yes/No) ...………… Type of Consent: (Verbal/Written) ……....………

		**Disagree**		**Neither**	**Agree**	
	**STATEMENT**	**-2**	**-1**	**0**	**+1**	**+2**
1	I think using B4M has made me more effective in my job. *(**Probe:** In what aspects of your job are you now more effective? Do you think it is the device that caused that?)*					
2	I think using B4M made it possible for me to attend to conduct ANC quickly. *(**Probe:** Is it important to you that you can quickly attend to clients? Why?)*					
3	Using B4M increased my workload *(**Probe:** How? All the time or only sometimes?)*					
4	With B4M, I am now able to do more things *(**Probe:** i.e. productivity. In what way? e.g. see more women, conduct tests and diagnose immediately, keep patient records easily. Do you feel you were less productive without B4M??)*					
5	With B4M I am now able to test patients properly for Anaemia, Pre-Eclampsia and Gestational Diabetes *(**Probe:** How did women get tested for PE, Anaemia & GDm before?)*					
6	It is easier to do ANC consultations now that I use B4M (***Probe:** What aspect of ANC services did you find difficult when you were not using B4M? Which part of B4M do you feel makes your work easier- px history, diagnosis, risk classification or px counselling?)*					
7	I think more pregnant women are coming for ANC now that we use B4M in the Health Facility *(**Probe:** Why do you think women were not coming for ANC before? How do you feel B4M solves that*					

	problem?)					
8	I think that the pregnant women follow my advice more now that I use B4M for consultation.<br>(***Probe:*** *Why do you think they comply more now?)*					
9	I do not trust the diagnosis that I get from the B4M device.<br>*(**Probe:** Why do you trust the diagnosis? What type of tests would you prefer?)*					
1<br>0	It is now easier for me to refer women that I identify as being at risk for Anaemia, Pre-Eclampsia and Gestational Diabetes.<br>*(**Probe:** Was this a difficulty in the past? In what way?)*					
11	I am not satisfied with using B4M for ANC consultations					
1<br>2	Using B4M improves my performance as a Health care worker<br>*(**Probe:** Can you give an example of when you felt your performance improved?)*					
1<br>3	Operating the B4M system is easy for me<br>*(**Probe:** What aspects of the device do your find difficult (e.g. typing, using the diagnostic sets, reading the traffic lights etc.))*					
1<br>4	The system is too complex for me to use<br>*(**Probe:** Are there any parts that you find easy or the entire system is complex?)*					
1<br>5	I am confident in my ability to use B4M<br>*(**Probe:** Do you think you are able to teach a new member of staff that has not been trained on how to use it?)*					
1<br>6	I think I need more training on how to use B4M<br>*(**Probe:** Do you want training on every aspect of the system or just some specific things?)*					
1<br>7	I like the traffic light signalling function of the decision-support<br>*(**Probe:** Show picture of the traffic lights. Ask midwife to tell you what the colours mean. What exactly does she like (or not like) about the lights function?)*					
1<br>8	The results and recommendations of the B4M confuses me<br>*(**Probe:** Can you remember a time when the system confused you? Give specific example.)*					
1<br>9	I find it easy to update information in the patients file using B4M					
2<br>0	It is difficult for me to do the Haemoglobin test using B4M					
2<br>1	It is easy for me to take blood pressure using B4M					

22	The urine glucose and protein tests are easy for me to do					
23	I usually need someone to assist me when I want to use the B4M system *(**Probe:** Who usually assists you when you need help?)*					
24	It is easier for me to use the usual system of ANC than with B4M (***Probe:*** *What (if any) are the advantages of the standard system in your opinion?)*					
25	What is the most positive aspect(s) of using B4M? *(audio recorded)*					
26	What is the most negative aspect(s) of using B4M? *(audio recorded)*					

Return to section 7 of the interview guide
