# Supplementary material for: Unveiling the Black Box of Diagnostic and Clinical Decision Support Systems for Antenatal Care: Realist Evaluation
Source: JMIR Mhealth Uhealth. 2018 Dec 21;6(12):e11468. doi: 10.2196/11468 (PMC6320439; doi:10.2196/11468)
Supplement: Multimedia Appendix 3 [file mhealth_v6i12e11468_app3.pdf]

Multimedia Appendix 3

CHECKLIST: FACILITY CAPACITY FOR PROVISION OF ANC SERVICES

Date ……………… Interviewer Initials …………... Location……………………

Health Facility Name…………………………………………………………..

	Yes	No	Irregular
**Equipment and Resources**			
Microscope			
Gloves and other disposables like cotton swabs			
Urine specimen bottles			
Sphygmomanometer + Stethoscope			
Pinnards Stethoscope			
Dipsticks for urinary protein and glucose			
Weight scale			
Height scale			
Measuring tape			
Transportation support for emergency referral			
Ultrasound			
ITNs for new registrants			
Electricity *(is it regular? is there backup for emergencies? What kind?)*			
**Drugs/Medication**			
Nifedipine			
$MgSo_4$			
Diazepam			
Tetanus Toxoid			
Antimalarial			
Folic Acid tablets			
Iron tablets			
**Which of these do you have full capacity to diagnose in this HF?** *If no, where do patients go for tests? Are tests free? If no, how much?*			
Pregnancy Test			
Rhesus Incompatibility			
Pre-eclampsia / Eclampsia			
Anaemia			
Gestational diabetes			
Malaria			
Urinary tract Infection			
**Which of these do you have the capacity to manage in this health facility?**			
Pre-eclampsia / Eclampsia			
Anaemia			
Gestational diabetes			
Malaria			
Urinary tract Infection			
**Do you have the capacity to take deliveries in health facility?** *At all times of the day and night?*			
