# Supplementary material for: Unveiling the Black Box of Diagnostic and Clinical Decision Support Systems for Antenatal Care: Realist Evaluation
Source: JMIR Mhealth Uhealth. 2018 Dec 21;6(12):e11468. doi: 10.2196/11468 (PMC6320439; doi:10.2196/11468)
Supplement: Multimedia Appendix 4 [file mhealth_v6i12e11468_app4.pdf]

Multimedia Appendix 4

Characteristics of B4M users and Usability survey scores

Health Facility [a]	Age	Cadre / Gender	Years of experience	Score, Perceived Usefulness	Score, Perceived Ease of use
**Low Usage Cluster**					
Facility B					
	31 years	Midwife / F	5 years	45	48
	35 years	Midwife / F	10 years	59	52
				52.0	***50.0***
Facility C					
	27 years	Midwife / F	4 years	54	56
	27 years	Enrolled Nurse[b] / F	2 years	54	59
	33 years	Midwife + Facility Manager / F	7 years	49	55
				52.3	***56.7***
Facility E	35 years	Health Assistant / F	10 years	54	44
	27 years	Enrolled Nurse[b] / F	2 years	49	39
	57 years	Community health nurse / F	32 years	-	-
				51.5	***41.5***
**Moderate Usage Cluster**					
Facility A					
	30 years	Midwife / F	5 years	47	56
	33 years	Midwife / F	9 years	52	55
	30 years	Midwife / M	5 years	46	46
				48.3	***52.3***
Facility D					
	24 years	Community health nurse / F	2 years	53	56
	50 years	Midwife + Facility Manager / F	28 years	53	60
				53.0	***58.0***
Facility F					
	28 years	Midwife / F	4 years	41	51
	53 years	Midwife / F	27 years	37	36
				39.0	***43.5***

[a] Facility A is the ANC unit of a district hospital and is the first level referral point for facilities B, C and D, which are health centers. Facility E is an independent public health unit of a district hospital, while F is a health center.

[b] An auxiliary cadre similar to health assistants
